# Palatal bone thickness for mini-implant placement in different skeletal facial patterns: A CBCT approach

**DOI:** 10.6026/973206300210635

**Published:** 2025-04-30

**Authors:** Ankita Das, Vikram Karande, Ameya Tripathi, Lincy Rachel Thomas, Zameer Pasha, Vaishali Malhotra, Ritik Kashwani

**Affiliations:** 1 Department of Orthodontics and Dentofacial Orthopedics, Rama Dental College, Hospital and Research Center, Kanpur, Uttar Pradesh, India; 2 Department of Oral & Maxillofacial Surgery, D.Y. Patil Dental School, Lohegaon, Pune, Maharashtra, India; 3 Department of Periodontology, Dental College Azamgarh, Uttar Pradesh, India; 4 Department of Orthodontics, AIMST University, Semeling, Bedong Kedah, Malaysia - 08100; 5 Consultant in Oral and Maxillofacial Surgery, Durrat Al-Alammi Dental Clinic, Al-Majmaah, Riyadh, Saudi Arabia; 6 Department of Prosthodontics and Crown & Bridge, Faculty of Dental Sciences, PDM University, Bahadurgarh, Haryana, India; 7 Department of Oral Medicine and Radiology, School of Dental Sciences, Sharda University, Greater Noida, India

**Keywords:** Cone beam computed tomography (CBCT), palatal bone, skeletal facial patterns

## Abstract

The thickness of the palatal bone perpendicular to the palatal curvature at various angles in different skeletal facial patterns using cone beam computed tomography (CBCT) is of interest to dentists. Hence, three groups (hyper-divergent, normo-divergent and hypo-divergent) based on the Frankfort mandibular angle and sagittal slices were taken at three and six-millimeter intervals along the mid-palatal suture. Transversal lines were created between specific teeth and bone thickness was measured at angles ranging from -30° to +30°. Data shows that the maximum bone thickness occurred at the first premolar and between the first and second premolars, with greater thickness in hyper divergent subjects. The anterior palate provided the best bone support for orthodontic mini-implants, with the greatest bone height found at the first premolar and paramedian regions.

## Background:

Movement of teeth can be done with Temporary Anchorage Devices is a compliance-free alternative to other traditional forms of anchorage used in orthodontics. Several studies have demonstrated their efficacy for in masse retraction, Class III therapy, space closure and many other applications for both adults and children [1]. According to Cheng *et al.*, although the preferred insertion site for mini-implants is the alveolar process, it still shows an average failure rate of 16.1% due to different bone and soft-tissue conditions. There are risks and complications of the use of TADs such as root perforation, perforation into the nasal and maxillary sinus and loss of implant due to inflammation [2].

The maxillary palate is a safe anatomical site for implant placement as it has sufficient bone quality, provides good accessibility and lowers the risk of damage to anatomical structures like roots, blood vessels etc. Several studies suggest that bone quality is superior within a T-shaped zone in the palate including the anterior palate and the median suture. To reduce the chance of perforations, the vertical bone height at the implant insertion location must be measured [3]. According to Becker *et al.* a posterior tipping was beneficial at anterior positions and an anterior tipping appeared beneficial at posterior positions. Therefore, the ideal insertion angle at different positions in the palate may be more clinically relevant [4]. Furthermore, none of the studies have compared the palatal bone height at different angulations in different vertical skeletal facial patterns. Because patients have different vertical facial patterns, this should also be taken into consideration in selecting the proper size and angulation of implant placement in the palate. Therefore, it is of interest to evaluate the thickness of the palatal bone perpendicular to the palatal curvature with angles varying from -30 degrees to +30 degrees at different positions within CBCT in different skeletal facial patterns.

## Materials and Methods:

This cross-sectional study included adult patients who had not undergone any previous orthodontic treatment. The exclusion criteria were the presence of any syndromes, malformations of bone, pathologic lesions in the maxilla and palatally displaced tooth. 33 CBCT of patients were and they were divided into 3 groups - Hyper-divergent (FMA> 29°)-12, Normo-divergent (FMA 21-29°)-10 and Hypodivergent (FMA <21°)-11. The CBCT images were stored in DICOM format and were analyzed using NNT viewer software. No informed consent was required as the CBCT scans were taken from the departmental archives. The CBCT scans were aligned according to the axial and sagittal planes and measurement positions were created through a series of steps. First, three and six-millimeter lateral sagittal slices along the mid-palatal suture were taken. Then, transverse reference lines perpendicular to the mid-palatal suture were drawn at the points of contact between the canines and first premolars (C-PM1), the first and second premolars (PM1-PM2) and the second premolar and first molar (PM2-M1), which allowed dental landmarks to be projected onto the measurement grid. Additional reference lines were constructed at the bicuspids by determining the midpoints of the intersections of the vectors from C-PM1 to PM1-PM2 and from PM1-PM2 to PM2-M1. A grid of 25 measurement points was created at the intersections of these sagittal and transverse reference lines and measurements were taken within five sagittal slices at the appropriate tooth projections. For each slice, the bony margin of each reference point was identified and measurements were taken perpendicular to the tangent at 0°, as well as at angles of -30°, -20°, -10°, 10°, 20° and 30°. The measurement tool was used to assess bone heights between the cortical boundary of the palate and the nasal or maxillary sinus, ending at appropriate anatomical positions if the line crossed tooth roots or the incisive canal.

## Statistical analysis:

Data obtained were compiled systematically in a Microsoft Excel spreadsheet. The dataset was subdivided and distributed meaningfully and presented as graphs and tables. The data was entered using MS Excel 2016. It was analyzed using IBM SPSS Statistics (Version 25). The continuous variables were presented in terms of Mean and Standard Deviation (SD), while the categorical variables were presented in terms of frequency and percentages. Inferential statistics for the correlation of the variables were done using One-way ANOVA statistics. The p-value was kept at 0.05. Measurements were done by one examiner to eliminate inter-examiner errors.

## Results:

Bone thickness at PM1 and PM1-PM2 was the maximum in all three groups. Paramedian bone heights were greater than median positions and it increased from C-PM1 to PM1 and then gradually decreased. They remained higher for anterior insertion angles at PM1-PM2 and then gradually decreased posteriorly. Anteriorly (C-PM1, PM1) the thickness was more for posterior insertion angulations. In the hyper-divergent group, bone height was maximum at 3 mm paramedian position (mean-12.42 ± 1.90mm) at PM1 (Figure 1 - see PDF). Whereas, in the median position, the maximum bone height was at PM1-PM2 region with posterior angulation. The mean was 6.7 ± 0.7mm (Figure 2 - see PDF). In the normo-divergent group, bone height was maximum at 6mm paramedian position (mean-11.04 ± 1.97mm) (Figure 3 - see PDF). In the median position, the maximum bone height was at the PM1-PM2 region with a mean of 6.82 ± 3.47mm (Figure 4 - see PDF). Bone height in hypodivergent group was maximum at 3mm paramedian position (mean- 6.79± 1.06mm) (Figure 5 - see PDF). At the median position, maximum bone height was at PM1-PM2 position (mean- 4.59 ± 1.02mm) (Figure 6 - see PDF). When comparing hyper-divergent, normo-divergent and hypo-divergent samples, the thickness of bone was greater in hyperdivergent subjects followed by normodivergent and hypo-divergent subjects had the least thickness of bone. An exception was present in the median plane where the thickness was greater in the normo-divergent subjects followed by hyperdivergent and then by hypodivergent subjects.

## Discussion:

The purpose of this study is to determine whether particular insertion angles are advantageous for orthodontic mini-implants in the anterior palate. Both the overall potential advantage of a certain angle and its specific paramedian and median placements were investigated. Then, based on the thickness of the bone, potential sites and angles for the implantation of orthodontic mini-implants were identified. Palatal bone thickness in various vertical skeletal patterns was assessed as a secondary outcome. A measurement grid was drawn into the palate to ascertain the bone thickness in various places. In previous research, the incisive foramen was chosen as a suitable anatomical landmark. However, frequently visualizing the foramen clinically is difficult. Therefore, in this study, premolar midline and interproximal tooth contacts were taken into consideration as landmarks. Our data supported prior findings that at the level of first premolars and between first and second premolars had the thickest bone and that the effective bone height decreased posteriorly. Effective bone heights peaked at the 3 mm and 6 mm paramedian positions, respectively and then dropped progressively posteriorly at both paramedian levels. The hyperdivergent group had a maximum bone height at a 3mm paramedian position (mean- 12.42 ± 1.90mm) followed by a 6mm paramedian position. In the normo-divergent group, maximum bone height was at 6mm paramedian position (mean- 11.04 ± 1.97mm). Whereas, in the hypodivergent group the maximum bone thickness was at 3mm paramedian position (mean- 6.79 ± 1.06mm). The result is consistent with earlier research by Benhart *et al.* [5] who found that 3-6 mm from the midline was the optimal position for implant insertion. According to King *et al.* [6] 3mm from the midline and 4mm from the incisive foramen was the best position for placement of the implant. According to research by Lai *et al.* [7], sites with adequate bone thickness are those that are 3 mm posterior to the incisive foramen and 6 mm from the median palate; 6 mm posterior to the foramen and 6 mm or 9 mm from the median palate. Bone thickness at the median region was lower than that of the paramedian regions. At this plane, the PM1-PM2 position had the greatest bone height in all the three groups. The mean bone height at the median region (PM1-PM2) for hyper-divergent, normo-divergent and hypo-divergent groups were 6.7 ± 0.7mm, 6.82 ± 3.47mm and 4.59 ±1.02mm respectively. The study by Baumgaertel *et al.* [8] which concluded that parasagittal placement of mini-implants at the level of the first and second premolars was best, found comparable results. This was in contrast to earlier research by Nakahara *et al.* [9] which found that the presence of the nasal crest caused bone thickness to rise in the median palate relative to the lateral regions. In our investigation, the risk of contracting the nasopalatine nerve was highest at places anterior to the first premolar, particularly in the median region. This agrees with research by Benhart *et al.* [5] that found the area near the incisive foramen to have the shortest mean value of bone thickness.

When comparing the different facial groups, although the results were statistically insignificant clinically the hyperdivergent group had more bone thickness followed by the normo-divergent and the hypo-divergent group except in the median plane, where, the normo-divergent group had more bone height followed by the hyperdivergent and hypodivergent groups. Implant placement in the hypo-divergent group should be done cautiously and pre-operative evaluation for the best possible location should be done. The maximum height of palatal bone (mean- 5.98 ± 2.41mm) for the hypodivergent group was at 3mm paramedian region (PM1). This was similar to the study done by Tavares *et al.* [10]. In contrast, Vidalon *et al.* [11] stated that individuals with hypodivergent facial forms had more bone thickness followed by normo-divergent and hyper-divergent groups. In the literature, various techniques to measure palatal effective bone height have been documented. Due to the superimposition of anatomical features, two-dimensional measurements made with the aid of lateral cephalograms are of little value. According to Wehrbein *et al.* [12] there is at least 2mm more vertical bone support than what is shown on lateral cephalograms. According to research by Wehrbein *et al.* [12] the amount of vertical bone seen on lateral cephalograms typically represents the minimal amount of bone and not the amount of vertical bone that exists in the mid-sagittal plane. Additionally, in the study, palatal heights in the median plane assessed using CBCT were significantly higher than those assessed using lateral cephalograms. As a result, it is routine practice to analyse CBCT pictures to assess the availability of bone in the anterior palate. Insertion angles and effective bone height have been assessed about a variety of reference planes, the majority of which have been the sagittal and coronal planes from CBCT. The study by Wang *et al.* [13] provides valuable insights into the variations in palatal bone thickness across different skeletal facial patterns, which is crucial for the successful placement of mini-implants. Their findings complement our CBCT-based approach, further emphasizing the importance of individualized treatment planning for mini-implant placement. Contrarily, we took slices either from the median plane or the paramedian plane. We thought that effective bone height in this direction was most pertinent for the doctor because implant implantation is advised to be done perpendicular to the palatal surface. Therefore, bone support after tipping the implant from -30° to the anterior to +30° to the posterior was also assessed. Implant placement perpendicular to the palatal surface was considered as the default 0° position.

## Conclusion:

The maximum bone height was found at the first premolar and between the first and second premolars, with the paramedian regions showing higher bone height than the median position, which extended to the second premolar. Anterior tipping of the implant was beneficial for paramedian and median implantations at posterior locations, while posterior tipping was more favorable for anterior median positions. When comparing the three groups, hyperdivergent subjects had the greatest bone height, followed by normo-divergent subjects, while hypo-divergent subjects had the least.
